# Present and Future of Autologous Breast Reconstruction: Advancing Techniques to Minimize Morbidity and Complications, Enhancing Quality of Life and Patient Satisfaction

**DOI:** 10.3390/jcm14082599

**Published:** 2025-04-10

**Authors:** Mario F. Scaglioni, Federica Martini, Matteo Meroni

**Affiliations:** 1 Plastic Surgery Pyramide, Haus zur Pyramide, 8008 Zurich, Switzerland; federicaa.martini@gmail.com (F.M.); matteomeronimd@gmail.com (M.M.); 2 Department of Health Sciences and Medicine, University of Lucerne, 6005 Lucerne, Switzerland; 3 Department of Hand- and Plastic Surgery, Luzerner Kantonsspital, 6000 Lucerne, Switzerland; 4 Department of Plastic, Reconstructive and Aesthetic Surgery, University of Milan, 20122 Milan, Italy

**Keywords:** autologous breast reconstruction, microsurgery, robotic surgery, lymphatic surgery, innovation

## Abstract

**Background:** Autologous breast reconstruction has undergone a remarkable evolution, driven by the pursuit of addressing past concerns primarily related to donor site morbidity and complication risks. Improved techniques now prioritize minimizing invasiveness, complications, and recovery time while achieving aesthetically pleasing and durable results. **Methods:** Recent advancements in autologous breast reconstruction have been examined, focusing on enhancements in surgical techniques, imaging technologies, minimally invasive approaches, and postoperative care. **Results:** To reduce donor site morbidity, attention has recently shifted back to abdominal flaps vascularized by subcutaneous vessels. Specifically, the superficial circumflex iliac artery perforator (SCIP) flap has emerged as a promising option. Additionally, robotic-assisted flap harvest serves as another method to reduce the invasiveness. At the recipient site, rib-sparing internal mammary vessel isolation and perforator-to-perforator anastomosis have been suggested to lessen trauma and maintain thoracic integrity. The use of thorough preoperative imaging and intraoperative assessment of real-time perfusion with indocyanine green angiography (ICG) has enhanced the success of the procedure. Beyond aesthetic restoration, contemporary breast reconstructive surgeons are increasingly aware of both short-term and long-term complications, particularly lymphatic sequelae. The LYMPHA technique (lymphatic microsurgical preventive healing approach) promotes immediate restoration of the lymphatic system and has shown the potential to reduce the risk of breast cancer-related lymphedema (BCRL). Furthermore, the integration of enhanced recovery after surgery (ERAS) protocols has transformed perioperative care by optimizing pain management, minimizing hospitalization duration, and allowing a quicker return to daily activities. **Conclusions:** Recent advancements in autologous breast reconstruction have significantly improved patient outcomes. With innovations in flap design, technology, lymphatic preservation, and recovery protocols, it has been possible to usher in a new era of less invasive procedures and fewer complications while achieving high aesthetic results and allowing patients to return to their daily lives as quickly as possible.

## 1. Introduction

Breast reconstruction is a crucial aspect of plastic surgery. For numerous women, undergoing a mastectomy can be a profoundly disfiguring experience, leading to emotional and psychological challenges [1]. Consequently, a primary objective for plastic surgeons is not only to reconstruct the breast shape but, more importantly, to restore the patient’s confidence and self-esteem. Among the available reconstruction techniques, autologous breast reconstruction has long stood out due to its ability to provide a natural breast shape with lasting results [2]. Initially, musculocutaneous flaps were the most commonly performed technique for breast reconstruction, with the transverse rectus abdominis myocutaneous (TRAM) flap [3] considered the gold standard. Although it was successful in reshaping the breast, this flap often led to significant issues at the donor site, including abdominal wall weakness and bulging or hernia development [4], prompting surgeons to reconsider their widespread use and find other solutions. To address these concerns and avoid rectus abdominis muscle harvest, a new solution was developed, and the deep inferior epigastric artery perforator (DIEP) flap [5] was proposed a few years later, ultimately becoming the gold standard for autologous breast reconstruction. Consequently, in order to further reduce abdominal morbidity and avoid opening the rectus fascia, the abdominal flap based on the superficial inferior epigastric artery (SIEA) was proposed [6]. Although it demonstrated reduced donor site morbidity and maintained abdominal wall integrity, its application remained limited due to the unpredictability of the vessels, which may occasionally be absent or hypoplastic. Additionally, it seemed to present a higher risk of pedicle kinking, shorter pedicle length, and reduced vascularization of the abdominal flap [7], resulting in less frequent use in previous years compared to the DIEP flap. Regarding other flaps proposed for autologous breast reconstruction, the profunda artery perforator [8] (PAP), transverse upper gracilis [9] (TUG), superior gluteal artery perforator [10] (SGAP), and the lumbar artery perforator [11] (LAP) flap, and others [12] represent valid alternatives. Moreover, autologous reconstruction, though an effective method, was often seen as a procedure associated with more risks, postoperative pain, extended surgical and recovery times, and complications. Regarding long-term complications, it is unfortunately common for patients who underwent axillary lymph node dissection (ALND) to develop breast cancer-related lymphedema (BCRL). This condition could severely impact a patient’s daily life, affecting mobility and overall quality of life. As a result, much more attention is now paid to the lymphatic system and its restoration after surgical damage, emphasizing the need for innovative solutions to manage or prevent this complication [13].

These evolving concerns have driven a shift towards more accurate tissue-sparing procedures. With advancements in surgical techniques and technology, options once considered impossible are now becoming a reality. This paper analyzes the most recent advancements in autologous breast reconstruction, exploring how these innovations have reduced invasiveness and complications while achieving high aesthetic results and allowing patients to return to their daily lives as quickly as possible.

## 2. Innovations to Minimize Morbidity and Complications in Autologous Breast Reconstruction

### 2.1. Expanding the Use of Perforator Flaps: Superficial Circumflex Iliac Artery Perforator (SCIP) Flap for Breast Reconstruction

**Figure 1:**
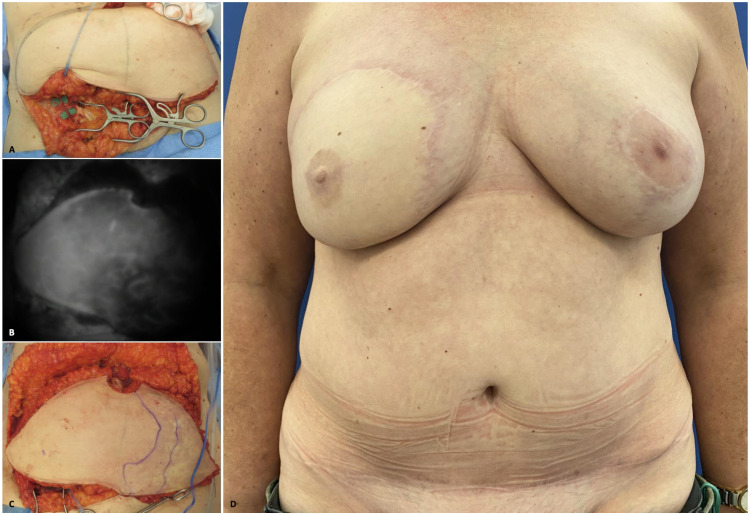
(**A**) Abdominal flap vascularized by the superficial branch of the superficial circumflex iliac artery (SB-SCIP); (**B**) intraoperative ICG imaging used to assess flap perfusion before vessels clipping; (**C**) marking after intraoperative ICG, indicating that perfusion crossed the midline; (**D**) postoperative image at the 12-month follow-up. (The images are derived from the senior author’s personal clinical practice).

The introduction of perforator flaps [14], which allow the preservation of muscles, harvesting the flap only on the source vessel, has revolutionized autologous reconstruction. The shift from traditional musculocutaneous flaps to perforator flaps has significantly decreased donor site morbidity and complications, marking an advancement in reconstructive surgery. The DIEP flap is still considered the gold standard; however, even though the donor site morbidity is generally low, other techniques have been proposed to further minimize it, such as laparoscopic DIEP flap [15] harvest and short fasciotomy DIEP [16], which aim to reduce the rectus fascia opening. Nonetheless, it still carries a risk of abdominal complications [17]. Recently, to further reduce donor site morbidity, there has been renewed interest in abdominal flaps vascularized by subcutaneous vessels. (Figure 1).

In particular, recent attention has focused on lower abdominal flaps supplied by the superficial branch of the superficial circumflex iliac artery (SB-SCIP flap) [18,19]. This technique, similar to the SIEA flap [6], offers a significant advantage by eliminating the need to open the rectus fascia and perform intramuscular dissection, thereby minimizing the risk of abdominal wall weakness and associated complications such as bulging or hernias. It also leads to reduced postoperative pain and faster recovery. An advantage compared to the SIEA flap is that the SB-SCIA, despite potential anatomical variations, is consistently present [20]. However, imaging modalities, such as computed tomography angiography (CTA), are essential to preoperatively visualize and localize the vessels. Intraoperative indocyanine green (ICG) angiography, in addition, plays a crucial role in ensuring adequate flap perfusion through the SB-SCIP vessels. However, despite its benefits, this flap presents notable technical challenges. The small-caliber perforating vessels may pose challenges for anastomosis with the internal mammary artery due to potential size mismatches, prompting surgeons to seek solutions to address the difference in caliber during the anastomosis. Additionally, it is crucial to harvest the flap with the superficial circumflex iliac vein. This vein has a larger caliber than the comitantes veins, which facilitates anastomosis with the internal mammary vein, improving venous drainage and reducing the risk of venous congestion in the flap. At the same time, the delicate dissection process requires precision to prevent vasospasm, which could result in false-negative ICG perfusion assessments [19].

### 2.2. Internal Mammary Artery Perforator-to-Perforator Anastomosis and Rib-Sparing Approach

**Figure 2:**
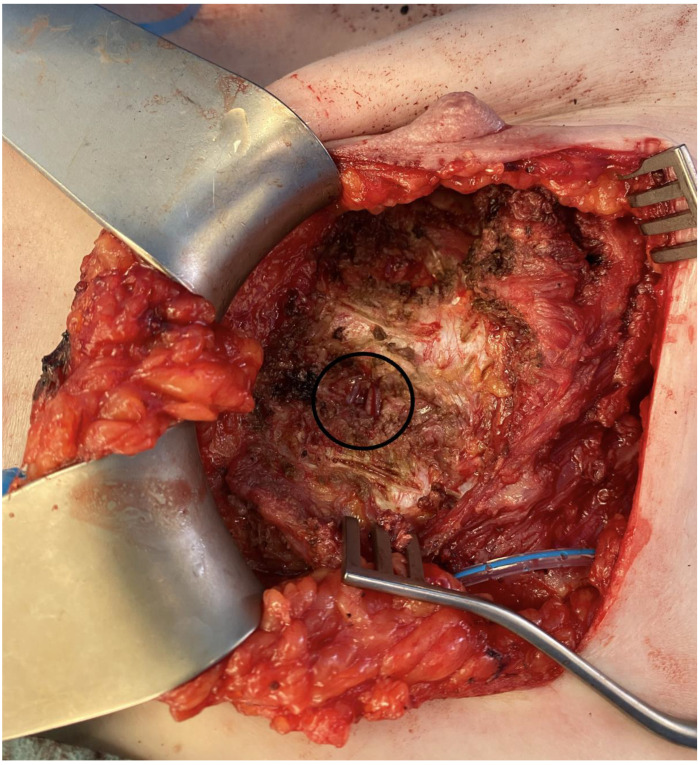
Rib-sparing exposure of internal mammary vessels. (The image is derived from the senior author’s personal clinical practice).

In autologous breast reconstruction, the internal mammary artery (IMA) is commonly selected as the recipient vessel for microvascular anastomosis [21]. Traditionally, exposure of the internal mammary vessels requires the resection of costal cartilage, which, although facilitating anastomosis by creating additional space, is associated with potential complications. These include an increased risk of vascular branch injury and pneumothorax during the perichondrium dissection from the vessels and pleura. Furthermore, patients may experience postoperative discomfort and pain due to chest wall deformity or sensory deficits resulting from this approach [22]. The rib-sparing technique offers a less invasive alternative, allowing vessel exposure without removing costal cartilage (Figure 2).

This efficient and safe method reduces operative time and minimizes chest wall morbidity and the risk of causing pneumothorax. However, it provides a more confined operative field, which may present challenges for the surgeon during anastomosis [22].

**Figure 3:**
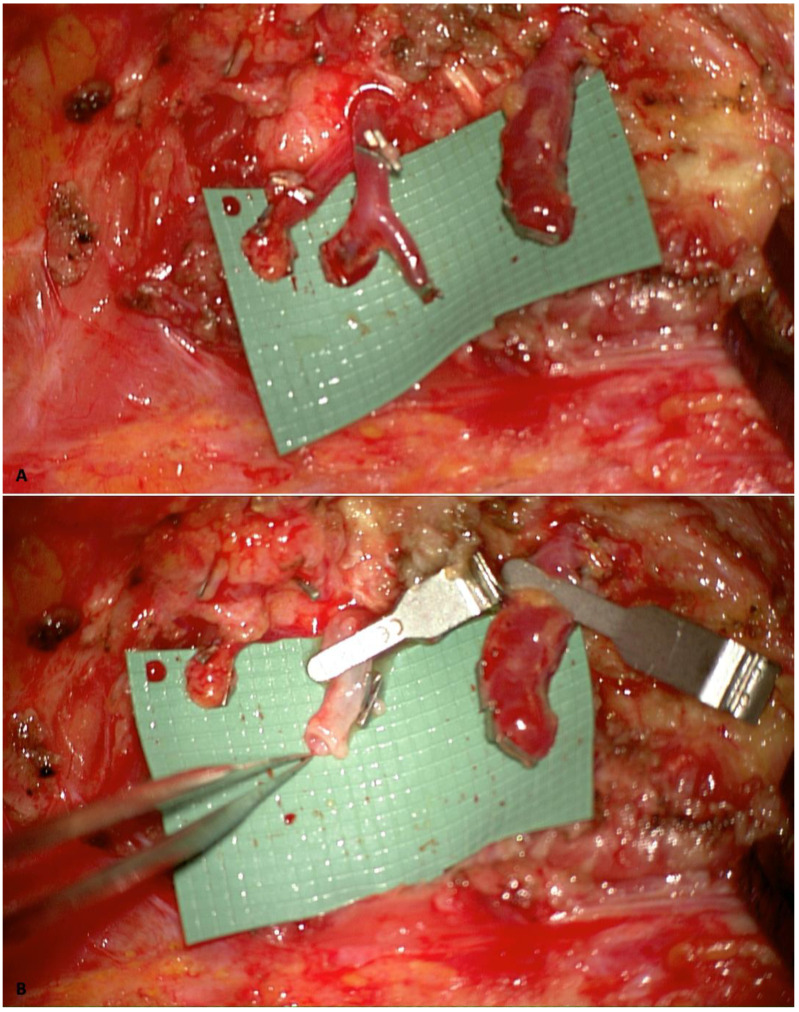
Image showing the Open-Y technique to enlarge the internal mammary artery perforator caliber to address vessel caliber mismatch. (**A**) IMAP isolated with a small branch, with a Y shape; (**B**) artery cut at the Y-bifurcation to widen the vessel’s caliber. (The images are derived from the senior author’s personal clinical practice).

Utilizing internal mammary artery perforators (IMAPs) [23] as recipient vessels is another viable option when suitable perforators could be preserved during the mastectomy. This approach facilitates anastomosis in a relatively flat surgical field and is associated with reduced morbidity since less dissection is required. Nevertheless, the smaller caliber of IMAPs can make anastomosis more technically demanding. It may result in a caliber mismatch with the flap’s vessels, requiring the surgeon to address it with different microsurgical techniques, such as the Open-Y technique [24] (Figure 3).

### 2.3. Indocyanine Green Fluorescence Angiography (ICG)

Indocyanine green fluorescence angiography has become an essential tool in microsurgical breast reconstruction, offering real-time assessment of tissue perfusion and improving surgical outcomes. This technique involves the intravenous injection of ICG dye, which binds to plasma proteins and is illuminated using near-infrared light, allowing for dynamic visualization of microvascular blood flow [25].

Indocyanine green angiography offers real-time feedback on perfusion, allowing surgeons to make intraoperative adjustments like revising the anastomosis or removing non-perfused areas of the flap, which can significantly influence the success of the procedure [26]. Recent studies highlight the systematic use of intraoperative ICG at multiple key stages, including after flap elevation, post-microsurgical anastomosis, immediately after flap inset, and following wound closure. Its application has been associated with decreased flap loss, reduced need for re-exploration, and improved surgical efficiency and safety by allowing for early detection of intraoperative vascular compromise, thus enabling immediate interventions [27].

Furthermore, ICG could assist in identifying and selecting the most reliable perforators during flap harvesting. By clamping the other perforators, it may be possible to visualize the flap perfusion based on the perforators the surgeon intends to use, with the ability to also assess perfusion from the other perforators and select the one that best vascularizes the flap [28,29]. Additionally, it aids in optimizing flap design by identifying areas with adequate tissue perfusion and removing poorly vascularized regions, thus reducing the risk of fat necrosis [30]. ICG angiography also plays a crucial role in evaluating mastectomy skin flaps, helping to reduce the risk of necrosis [31]. With its growing adoption, ICG fluorescence angiography continues to refine microsurgical breast reconstruction by enhancing safety, precision, and overall success rates.

### 2.4. Preoperative Imaging Evaluation

Preoperative imaging evaluation has become an essential step in autologous breast reconstruction surgery, helping surgeons carefully plan the procedure, perform it more efficiently, and improve surgical outcomes. The primary goal is to thoroughly analyze the patient’s vascular anatomy, allowing the surgeon to evaluate the suitability of the patient for the procedure and rule out the possibility of vascular anomalies that could potentially determine the failure of the procedure. Moreover, in the setting of perforator flap reconstruction, it enables surgeons to accurately select in advance from which side is more advantageous to harvest the flap, identify the dominant perforators, and, according to its location, improve the design of the flap, ensuring that the vessel will be localized in the central portion of the flap to enhance the perfusion. Making all these decisions preoperatively greatly contributes to reducing operative time and improving surgical efficiency since the surgeon can speed up the dissection up to the area close to the selected vessel and then slow it down to perform a careful vessel dissection [32].

Among the various techniques, computed tomography angiography (CTA) is considered the gold standard. It provides high-resolution and accurate images of blood vessels, giving surgeons a clear, detailed, and reliable view of the course of the selected vessel. In the setting of the DIEP flap, taking the umbilicus as a landmark, the location of the vessel could be calculated and reported to the patient. Additionally, it can reveal incidental findings, such as abnormalities in nearby blood vessels or in other anatomical regions analyzed during the imaging study. However, CTA exposes the patient to radiation and requires contrast dye, which can pose risks for those with kidney problems or contrast allergies [32].

Another alternative is magnetic resonance angiography (MRA). Using gadolinium-based contrast agents offers high-quality vascular imaging. Moreover, it gives excellent soft-tissue contrast without the use of ionizing radiation, making it a safer option. Regarding the drawbacks, it takes longer to perform, has higher costs, and is not suitable for patients with metal implants, gadolinium allergies, or claustrophobia [33,34].

Furthermore, even with the benefits of CTA and MRA, handheld Doppler ultrasound and color Doppler ultrasound remain widely utilized tools for localizing blood vessels prior to surgery. Their continued use is attributed to their affordability, portability, and easy accessibility. Additionally, they are non-invasive, cost-effective, and free from radiation, which makes them valuable for real-time vessel mapping and blood flow evaluation. However, they have limitations, as their accuracy depends on the experience of the person using them, and they do not provide a full three-dimensional picture of the vascular network [34].

Artificial intelligence (AI) is also suggested as a prompting support tool for preoperative image analysis and thus assists in surgical planning. Given that analyzing images and developing a surgical plan can be time-consuming and influenced by the surgeon’s experience, utilizing AI in this process might become advantageous. Although further refinements are needed, AI shows potential for broad future application, particularly in facilitating image analysis and perforator identification; thus, it may contribute to greater precision and efficiency [35].

### 2.5. Robotic-Assisted Surgery

Robotic-assisted surgery is revolutionizing how surgeons perform flap harvesting, offering greater precision, less trauma, and better patient outcomes. By eliminating natural hand tremors and enabling extremely fine movements, robotic systems allow for smaller incisions and minimal disruption to surrounding tissues [36]. This is especially beneficial in autologous breast reconstruction, where delicate procedures like DIEP flap harvesting require meticulous dissection of the vascular pedicle while minimizing trauma to the rectus abdominis muscle and avoiding motor nerve interruption.

Research has shown that robotic-assisted flap harvesting leads to less postoperative pain, shorter hospital stays, and faster recovery times [36,37]. A systematic review and meta-analysis further highlighted its role in improving surgical precision, particularly in DIEP and latissimus dorsi (LD) flap harvesting, leading to reduced postoperative complications and better aesthetic outcomes [37]. Beyond flap harvest, robotic technology is now being explored in supermicrosurgical procedures, including the precise anastomosis of blood and lymphatic vessels smaller than 0.8 mm in diameter [38]. This development presents significant opportunities, enabling even less experienced surgeons to anastomose small vessels and carry out complex reconstructions effectively. As a result, conditions such as lymphedema may receive broader treatment, as robotic-assisted lymphaticovenular anastomosis could increase the number of facilities and surgeons capable of offering this therapy, ultimately enhancing the quality of life for lymphedema patients.

However, like any emerging technology, robotic surgery comes with its own set of challenges. The biggest obstacle is cost since robotic systems require a significant financial investment, making them inaccessible to many hospitals. Additionally, robotic procedures often take longer than traditional methods due to setup time and intraoperative adjustments. There is also a steep learning curve, meaning surgeons must undergo extensive training to fully master the technology. These factors limit the widespread adoption of robotics in breast reconstruction despite its clear benefits [36].

Nonetheless, the future of robotic-assisted surgery appears highly promising. As the technology continues to advance and becomes more cost-effective, more hospitals and surgical centers will likely adopt it. Ongoing enhancements in robotic training programs will increase surgeons’ proficiency, making robotic-assisted surgery a potential standard in reconstructive procedures within the following years. This advancement is expected to facilitate quicker recoveries, minimize complications, and improve long-term patient outcomes.

### 2.6. Buried Flaps: Innovation in Flap Monitoring

In breast reconstruction, it is common practice to leave a skin flap island for monitoring purposes, and it could be particularly beneficial in delayed procedures where additional skin is often necessary. However, the need for extra skin is reduced with the growing prevalence of skin-sparing and nipple-sparing mastectomies [39]. In these cases, the skin island is often retained solely for clinical monitoring of flap perfusion. While effective for postoperative assessment, this approach, especially in the setting of nipple-sparing mastectomy, can compromise the aesthetic appearance of the reconstructed breast and frequently necessitates a second surgery to remove the excess skin and refine the breast contour.

For cases where only breast volume restoration is required, a fully de-epithelialized, buried flap may offer a superior alternative [40]. This technique not only enhances the aesthetic outcome and improves patient satisfaction [41] but also reduces the need for secondary reconstructive procedures, minimizing the risk of “reconstructive burnout” [42] and facilitating a smoother recovery process with a faster return to daily life for patients.

However, a key concern with buried flaps is the inability to assess perfusion through direct clinical observation. Without alternative monitoring techniques, potential vascular complications may go undetected until it is too late. To address this challenge, technological advancements have introduced reliable devices for monitoring anastomotic patency and flap viability in buried reconstructions.

One such device is the venous flow coupler [43,44,45], which serves a dual purpose: facilitating venous anastomosis and enabling continuous venous flow monitoring via an external wire. This wire, which connects to a monitoring unit, can be easily removed with gentle traction before the patient is discharged. One drawback of this approach is that it can measure only venous flow, not arterial flow. Nevertheless, venous insufficiency is still the primary cause of flap failure [46]. Additionally, there could be complications with the external wire since it may be unintentionally pulled, resulting in altered monitoring.

Another method is the use of implantable Doppler devices [47]. These systems rely on a pulsed ultrasonic Doppler probe stabilized around a target vessel using a silicone cuff. The probe is connected to an external monitor via a thin wire that exits through the surgical site. It could be used for either arterial or venous monitoring, providing continuous real-time feedback on vascular status and offering a crucial safety measure for buried flaps. The drawbacks include the ability to assess vascular occlusion only in the vessel to which the device is attached. Additionally, while the wire is removed before discharge, the silicone cuff remains on the vessel. Rarely, removal of the wire can cause traction on the vessel, leading to potential bleeding. The wire is also at risk of malposition; any inadvertent traction could misplace the device, resulting in cessation of the flow monitoring that could be interpreted as a vascular occlusion [48].

Near-infrared spectroscopy (NIRS) [49,50] has also emerged as a promising tool for flap perfusion monitoring. This non-invasive method assesses tissue oxygenation and perfusion by measuring hemoglobin oxygen saturation levels using near-infrared light. Recent studies suggest that NIRS may also be beneficial for buried flap monitoring [51,52]. Another advantage of this system is its minimal impact on patient mobility during recovery, as it allows temporary disconnection from the monitor, enabling patients to move more freely. One drawback is that the sensors adhere to the skin with glue or stickers, requiring frequent repositioning. Additionally, fluctuating SpO_2_ and blood pressure may affect the accuracy of the measurements. Furthermore, while uncommon, thrombosis might go undetected due to the presence of a hematoma [52].

Additionally, a common drawback of these devices is their cost, as implementing such technology requires substantial investment in equipment and staff training. Moreover, the decision to use this device often depends on the surgeon’s personal preference and experience. Many surgeons still favor the traditional clinical evaluation of the flap, relying on their expertise to assess perfusion and viability. Additionally, in other surgical scenarios, such as skin-sparing mastectomy or skin-reducing mastectomy, even if extra skin is not required, leaving a small skin island could benefit both flap monitoring and future reconstruction. Specifically, when the nipple-areola complex (NAC) is removed, placing the flap’s skin island in the area previously occupied by the NAC can provide a foundation for initial areola reconstruction, which facilitates secondary nipple reconstruction or NAC tattooing at a later stage. Consequently, it is crucial to tailor the best approach carefully based on the patient’s individual characteristics, reconstructive goals, and the surgeon’s personal expertise and preferences.

### 2.7. Lymphatic Complications Prevention

Breast cancer-related lymphedema [13] remains a significant and often debilitating complication for patients, profoundly affecting their physical well-being and overall quality of life. This condition, characterized by the accumulation of lymphatic fluid due to impaired drainage following surgical intervention, can lead to persistent swelling, discomfort, restricted mobility, and an increased risk of skin infections.

Recent advances in lymphatic surgery have been driven by a deeper understanding of lymphatic anatomy and physiology [53], along with remarkable progress in microsurgical techniques and imaging technologies. These developments have led to minimally invasive procedures, such as lymphaticovenous anastomosis (LVA) [54], which offer new treatment options for patients with established lymphedema or its prevention. By connecting functional lymphatic vessels to nearby veins before the obstruction, LVA reroutes lymph flow, bypassing the blockage and allowing for lymphatic fluid drainage directly into the venous system, reducing swelling and improving patient symptoms.

**Figure 4:**
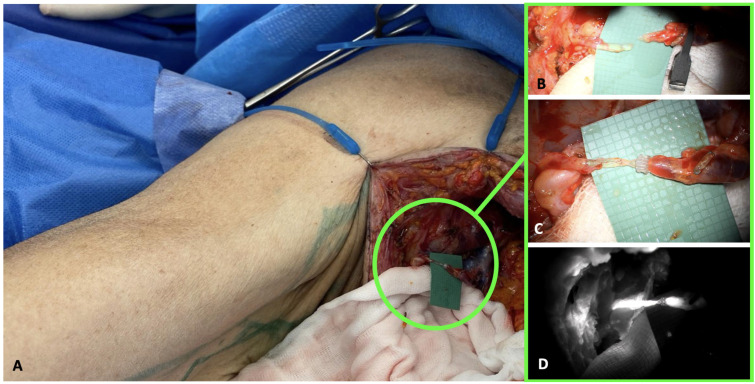
(**A**) Surgical field following axillary lymph node dissection, showing the anastomosis of a lymphatic vessel draining the upper limb with a branch of the axillary vein; (**B**) intraoperative view of the lymphatic vessel and the vein before the LYMPHA; (**C**) intraoperative image after completion of the preventive LVA with employment of a coupler device; (**D**) intraoperative ICG imaging confirming LVA functionality. (The images are derived from the senior author’s personal clinical practice).

A significant milestone in this field is the introduction of the LYMPHA (lymphatic microsurgical preventive healing approach) technique proposed by Boccardo et al. [55], which presents an effective strategy for preventing secondary lymphedema following axillary lymph node dissection. The procedure involves performing an immediate LVA at the time of lymph node removal between the damaged lymphatic vessels of the upper arm and a collateral branch of the axillary vein, creating a new drainage pathway for the lymphatic flow of the upper arm (Figure 4).

This allows for restoring physiological lymphatic flow, potentially reducing the risk of the manifestation of BCRL.

Despite its promising results, LYMPHA has not yet been widely adopted across medical centers. The primary challenges include the lack of high-level clinical evidence supporting its long-term efficacy and the technical expertise required for its successful execution. Microsurgeons must possess advanced skills to precisely identify and anastomose fragile lymphatic vessels, making it a resource-intensive procedure that is not universally available [13].

Nevertheless, studies have demonstrated encouraging results, with LYMPHA significantly lowering the incidence of postoperative lymphedema. Its potential extends beyond breast cancer surgery, as similar preventive lymphatic interventions have shown promise in reducing lymphedema risk in other oncologic procedures [56]. With continued advancements in imaging, surgical techniques, and clinical research, LYMPHA may become a widely accessible and essential component of modern breast cancer care, reducing the incidence of this complication and improving the quality-of-life outcomes for breast cancer patients.

### 2.8. Enhanced Recovery Protocols

The implementation of enhanced recovery after surgery (ERAS) protocols has significantly transformed perioperative care in autologous breast reconstruction. Integrating evidence-based practices, it optimizes surgical outcomes through a multidisciplinary approach that includes several key components. Among these are multimodal analgesia, reduced opioid use, early mobilization, and personalized patient education [57]. These strategies not only shorten hospital stays but also reduce the risk of nosocomial infections and thromboembolic events. Additionally, they accelerate recovery, enabling patients to return to daily activities more quickly, which enhances their psychological well-being and improves overall outcomes, making ERAS a key component in modern breast reconstruction practices [58].

## 3. Discussion

Autologous breast reconstruction has seen significant advancements, primarily driven by continuous improvements in surgical techniques, a deeper understanding of flap anatomy and physiology, and the ongoing evolution of medical technology. These enhancements have allowed surgeons to achieve high success rates and more natural results that more effectively meet patients’ expectations. The emphasis on minimizing surgical invasiveness and enhancing procedural efficiency has become a key goal in contemporary surgery. In modern autologous breast reconstruction, the focus is not only on achieving high-quality, aesthetically pleasing, and long-lasting results but also on reducing donor and recipient site morbidity, shortening operative times, minimizing complication rates, and improving recovery processes. The advancements discussed in this paper have been crucial in effectively addressing previous concerns related to donor and recipient-site morbidity, operative complexity, postoperative complications, and recovery. The integration of advanced technologies for more thorough and accurate preoperative evaluation and surgical planning, along with the use of intraoperative tools for assessing flap perfusion, has significantly improved both success rates and flap survival. For instance, imaging techniques such as CTA and MRA [32,33,34] allow surgeons to thoroughly assess the patient’s vascular anatomy preoperatively, identify any anatomical variations, and adjust the surgical plan accordingly. Intraoperatively, the use of indocyanine green angiography [25,26,27,28,29,30] has emerged as an essential tool that provides real-time assessment of blood flow, allowing surgeons to quickly detect compromised anastomoses or poorly vascularized areas. Early identification and correction of these issues significantly reduce the need for secondary revision surgeries and minimize the risk of complications, leading to better overall patient outcomes. The use of less invasive surgical techniques for flap harvesting [15,16,17,18,19] and recipient vessel isolation [22,23] or employing robotic technology [36,37,38] further decreases surgical morbidity. These approaches not only reduce postoperative pain and enable quicker recovery times but also make the overall process safer and more effective. Furthermore, enhanced perioperative protocols [57,58] and innovative flap monitoring techniques [43,44,45,47,48,49,50,51,52] have significantly contributed to facilitating postoperative patient management, enhancing patient recovery and satisfaction. Continual refinements of techniques and technology have enabled surgeons to perform these procedures more safely and efficiently, resulting in shorter surgery times and thus reducing the risks of complications associated with prolonged operation times.

Nevertheless, despite these achievements, significant challenges remain. The long learning curve of microsurgical procedures and the need for surgical expertise continue to limit the widespread adoption of autologous reconstruction. Moreover, the advanced technology required to enhance surgical precision, improve safety, and increase efficiency in these procedures is often associated with significantly higher costs. As a result, such technologies may not be available in all healthcare facilities, which can limit the widespread adoption of these techniques. This lack of accessibility may also increase the risk of complications when alternative, less advanced methods are used. Continued research and innovation are crucial to further refining current surgical approaches. Additionally, efforts should focus on expanding training programs and developing cost-effective and widely accessible solutions to ensure that more patients can benefit from these high-quality breast reconstructions. By pushing the boundaries of surgical innovation, the future of autologous breast reconstruction lies in achieving safer, more efficient, and less invasive techniques, ultimately improving patient outcomes and quality of life. The main limitation of this paper is that it was not designed as a systematic review; therefore, some relevant articles or recent innovations may have been overlooked. Additionally, the aim was not to provide a comprehensive, detailed description of all available techniques and technologies but rather to present an overview of advancements.

## 4. Conclusions

The advancements in autologous breast reconstruction have significantly improved surgical outcomes by enhancing procedural efficiency and safety, reducing morbidity, and achieving high-quality, natural results. However, challenges remain. Therefore, continued research, technological advancements, and improved training programs are essential to making autologous reconstruction safer, more efficient, and more widely available. Future progress will further enhance patient outcomes, making high-quality breast reconstruction accessible to a broader range of patients.

## Data Availability

The original contributions presented in this study are included in the article. Further inquiries can be directed to the corresponding author(s).
